# Enhanced migration and adhesion of peripheral blood neutrophils from SAPHO patients revealed by RNA-Seq

**DOI:** 10.1186/s13023-019-1169-3

**Published:** 2019-08-08

**Authors:** Yuxiu Sun, Chen Li, Mengmeng Zhu, Shen Zhang, Yihan Cao, Qiao Yang, Pengfei Zhao, Guangrui Huang, Anlong Xu

**Affiliations:** 1 School of Life Sciences, Beijing University of Chinese Medicine, Beijing, China; 2 Department of Traditional Chinese Medicine, Peking Union Medical College Hospital, Peking Union Medical College and Chinese Academy of Medical Sciences, Beijing, China; 3 Department of Radiology, Peking Union Medical College Hospital, Peking Union Medical College and Chinese Academy of Medical Sciences, Beijing, China; 4 Department of Nephrology, Peking Union Medical College Hospital, Peking Union Medical College and Chinese Academy of Medical Sciences, Beijing, China; 5 State Key Laboratory of Biocontrol, Department of Biochemistry, School of Life Sciences, Sun Yat-Sen (Zhongshan) University, Guangzhou, Guangdong China

**Keywords:** SAPHO syndrome, RNA-Seq, Neutrophil, Cell adhesion, Cell migration

## Abstract

**Background:**

SAPHO syndrome is a rare disease characterized by inflammatory lesions on skin and bones. Diversified manifestation and inadequate understanding of etiology has limited its diagnosis and treatment. The co-occurrence of other immune-mediated diseases strongly suggests an involvement of autoimmunity in SAPHO syndrome. However, the role of the largest population of circulating immune cells, neutrophils, is still not well explored. In this study, we performed RNA sequencing to profile the mRNA expression of neutrophils purified from peripheral blood of SAPHO patients to identify key genes associated with SAPHO syndrome, trying to find new functional molecules or biomarkers for this rare disease.

**Results:**

A total of 442 differentially expressed genes were identified (*p* < 0.05, fold change > 2), in which 294 genes were upregulated and 148 genes were downregulated. Five differentially expressed genes of interest were verified by quantitative Real-Time Polymerase Chain Reaction (qRT-PCR), among which *S100A12* was upregulated and positively related to high-sensitivity C-reactive protein (hsCRP), while the downregulated gene *MYADM* was positively related to osteocalcin. Kyoto Encyclopedia of Genes and Genomes (KEGG) analysis showed that differentially expressed genes were enriched in “systemic lupus erythematosus” and “ECM-receptor interaction”. Gene ontology (GO) enrichment showed that differentially expressed genes may participate in biological processes such as “cell migration” and “cell adhesion”.

**Conclusions:**

In conclusion, this study provides a first insight into transcriptome characteristics of SAPHO syndrome, indicating an over-active neutrophil recruitment in patients and possibly suggesting molecular candidates for further study on diagnosis and pathology of this disease.

**Electronic supplementary material:**

The online version of this article (10.1186/s13023-019-1169-3) contains supplementary material, which is available to authorized users.

## Introduction

SAPHO syndrome is a relatively rare disease known under the acronym of SAPHO (Synovitis, Acne, Pustulosis, Hyperostosis and Osteitis), which was first named by French rheumatologists in 1987 [1]. It is characterized by a wide range of dermatological and osteoarticular lesions, involvement of characteristic target sites (such as anterior chest wall, axial skeleton and pelvic bones [2]), and a clinical course of recurrently relapses and remissions without specific histopathologic features [3]. Lacking of validated molecular diagnostic criteria, the diagnosis of SAPHO syndrome is challenging and mainly based on clinical and radiological findings [4]. The fact that dermatological and osteoarticular lesions may occur many years apart or even not both present on the same patient always leads to delayed diagnosis, with patients suffering ongoing pain and skin manifestations [5].

Although the pathogenesis of SAPHO syndrome largely remains unknown, the significantly exhibited overlap and co-occurrence of manifestations of other immune-mediated diseases such as ankylosing spondylitis, psoriasis vulgaris and inflammatory bowel disease (IBD) [6] strongly suggests an inflammatory response, possibly involving autoimmunity [7]. Several previous studies have explored the role of immune cells in the development and prognosis of SAPHO syndrome. For instance, natural killer cells were found to be significantly reduced [8], while peripheral Th17 cells were reported to be increased in patients with SAPHO syndrome [9]. As for the largest population of innate immune cells, neutrophils, existing evidence has been limited. Although enhanced neutrophil infiltration was observed in PSTPIP2-deficient mice which presented a SAPHO syndrome-like phenotype [10], no significant increase in serum neutrophils in patients had been found. Neutrophils purified from patients with SAPHO syndrome were reported to have an abnormal internal oxidant production following stimulation with phorbol myristate acetate (PMA) [11], but no signs of aberrant intercellular oxidant production was shown [12]. Until recently, there has not been further investigation on the role of neutrophils in SAPHO syndrome. Meanwhile, it has been well understood that neutrophils play multifaceted roles in autoimmune diseases [13] and neutrophil-derived chemokines contribute to the pathogenesis of non-infectious diseases [14]. Thus, our study focused on the differentially expressed mRNAs in peripheral neutrophils from patients with SAPHO syndrome, trying to descript how the most abundant leucocytes, as well as one of the earliest immune cells to arrive at the inflammatory sites in the human body participate in SAPHO syndrome.

Genome-wide RNA sequencing (RNA-Seq) and bioinformatics analysis have been widely applied in clinical and basic research to identify key mRNAs that regulate autoimmune diseases [15, 16]. However, the molecular function and clinical value of mRNAs profile in SAPHO syndrome are still not explored. In this study, we performed RNA-Seq and bioinformatics analysis to identify key mRNAs associated with SAPHO syndrome, trying to find new functional molecules or biomarkers for this rare disease.

## Results

### Clinical features of included individuals

Six SAPHO patients and six healthy volunteers were recruited between March 2017 and June 2018. The historical disease status and clinical manifestation of each patient are shown in Table 1. All six patients suffered from bone lesions, with five on anterior chest wall, six on axial skeleton, two on peripheral joints and one on mandible. Skin involvement was observed in five patients, among which palmoplantar pustulosis is most commonly presented (5/6). Patient #1 was the only one without cutaneous lesions, whose osteoarticular manifestations were similar to the others except for an additional involvement of mandible. As shown in Table 2, there was no significant difference in age or gender between SAPHO group and Control group. Although the levels of peripheral white blood cells and neutrophils are slightly higher in patients with SAPHO syndrome, no statistical significance was found (*p* = 0.101 and *p* = 0.117, respectively). The mean visual analogue scale (VAS) of recruited patients is 4.50, indicating a middle-to-severe pain of infected bones. Compared with healthy controls, high-sensitivity C reactive protein (hsCRP) is slightly higher in patients. Besides, three of the patients exhibited faster erythrocyte sedimentation rate (ESR), while two had a lower level of serum osteocalcin, although the average levels of ESR and osteocalcin are within the normal range.

**Table 1:** Disease status and clinical manifestation of each patient

Patients #	Disease duration (months)	Clinical manifestation	
		Skin	Bone (affected part)
1	10	none	anterior chest wall, axial skeleton, mandible
2	108	Palmoplantar pustulosis	anterior chest wall, axial skeleton
3	6	Palmoplantar pustulosis	axial skeleton, peripheral joint
4	7	Palmoplantar pustulosis	anterior chest wall, axial skeleton, peripheral joint
5	6	Palmoplantar pustulosis	anterior chest wall, axial skeleton
6	24	Palmoplantar pustulosis, psoriasis vulgaris	anterior chest wall, axial skeleton

**Table 2:** Demographics and clinical parameters of included individuals

	Control	SAPHO	Reference range	*p* value
Gender	female	female		1
Age	25.33 ± 1.75	33.16 ± 10.26		0.053
White Blood Cell (*10^9^/L)	5.63 ± 0.95	7.27 ± 2.02	4.0–10.0	0.101
Neutrophil (*10^9^/L)	3.00 ± 0.81	4.24 ± 1.57	1.8–6.3	0.117
VAS (1–10)	–	4.50 ± 2.59	0–10	–
hsCRP (mg/L)	–	3.03 ± 3.63	0–3	–
ESR (mm/h)	–	15 ± 8.67	0–20	–
Osteocalcin (ng/L)	–	3.73 ± 1.83	1.8–8.4	–

### Overview of RNA-Seq

**Fig. 1:**
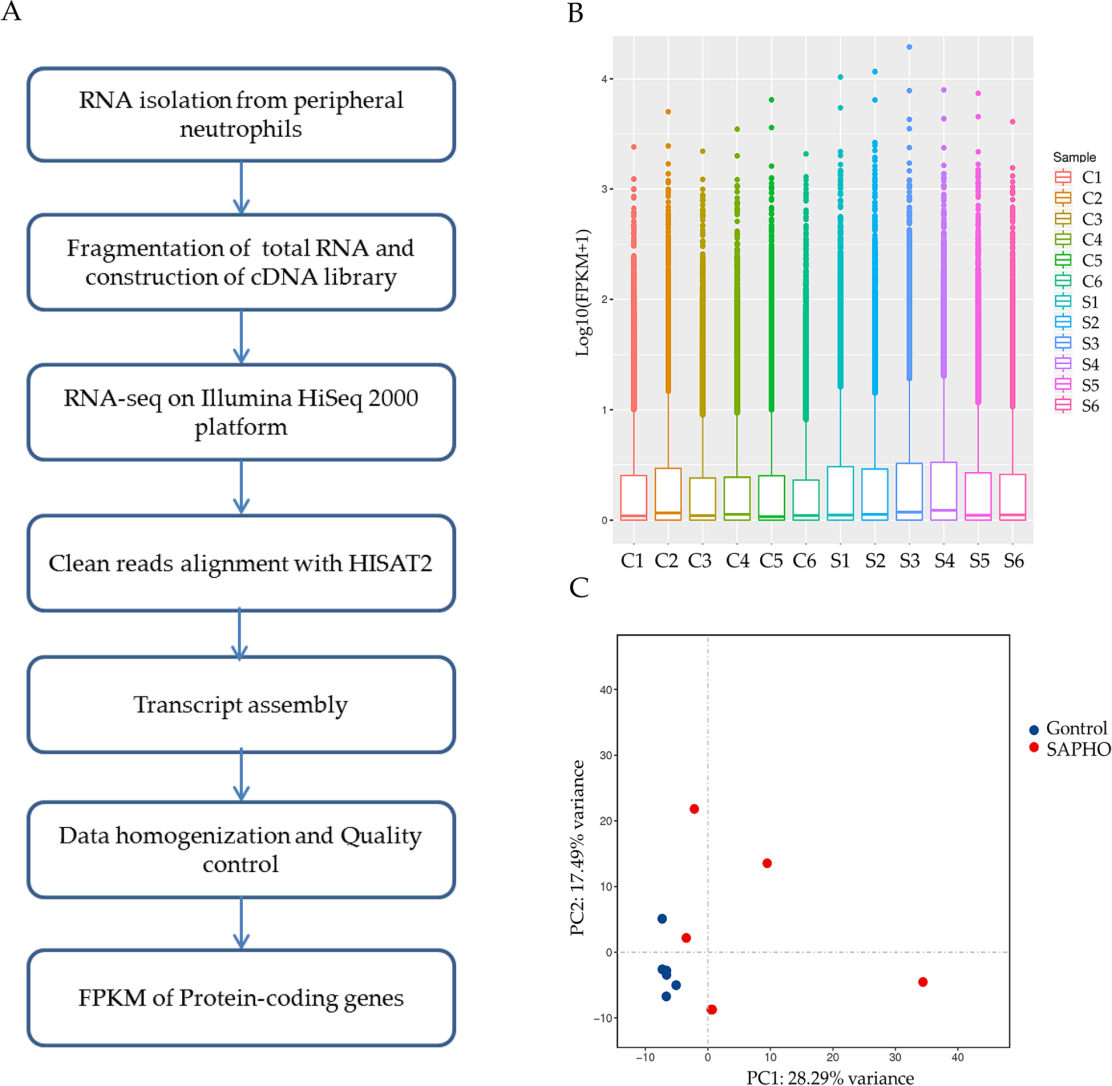
Overview of RNA-Seq. **a** RNA-Seq procedure. **b** Boxplot for FPKM values of all transcripts in each sample. **c** PCA map of all samples in Control and SAPHO group

To systematically analyze mRNAs related to SAPHO syndrome, approximately 1.15 billion reads for 12 samples from 6 patients and 6 healthy controls were sequenced using RNA-Seq (Additional file 1: Table S1). Experimental and computational scheme for the RNA-Seq process is shown on Fig. 1a. Firstly, the total RNA from neutrophils was isolated and reverse-transcribed into cDNA library. RNA sequencing was then performed on Illumina HiSeq 2000 Platform. Clean data was mapped to GRCh38 with HISAT2 [17] and then assembled into transcripts. After data homogenization and quality control, the expression level of each transcript was calculated by fragments per kilobase of exon per million fragments mapped (FPKM). The boxplot of FPKM values of all transcripts indicated similar whole transcriptome expression in each sample (Fig. 1b) and principal component analysis (PCA) revealed a relatively clear distinction between samples in the two groups along the first principal component of 28.29% variance (Fig. 1c).

### Differentially expressed genes in SAPHO patients

**Fig. 2:**
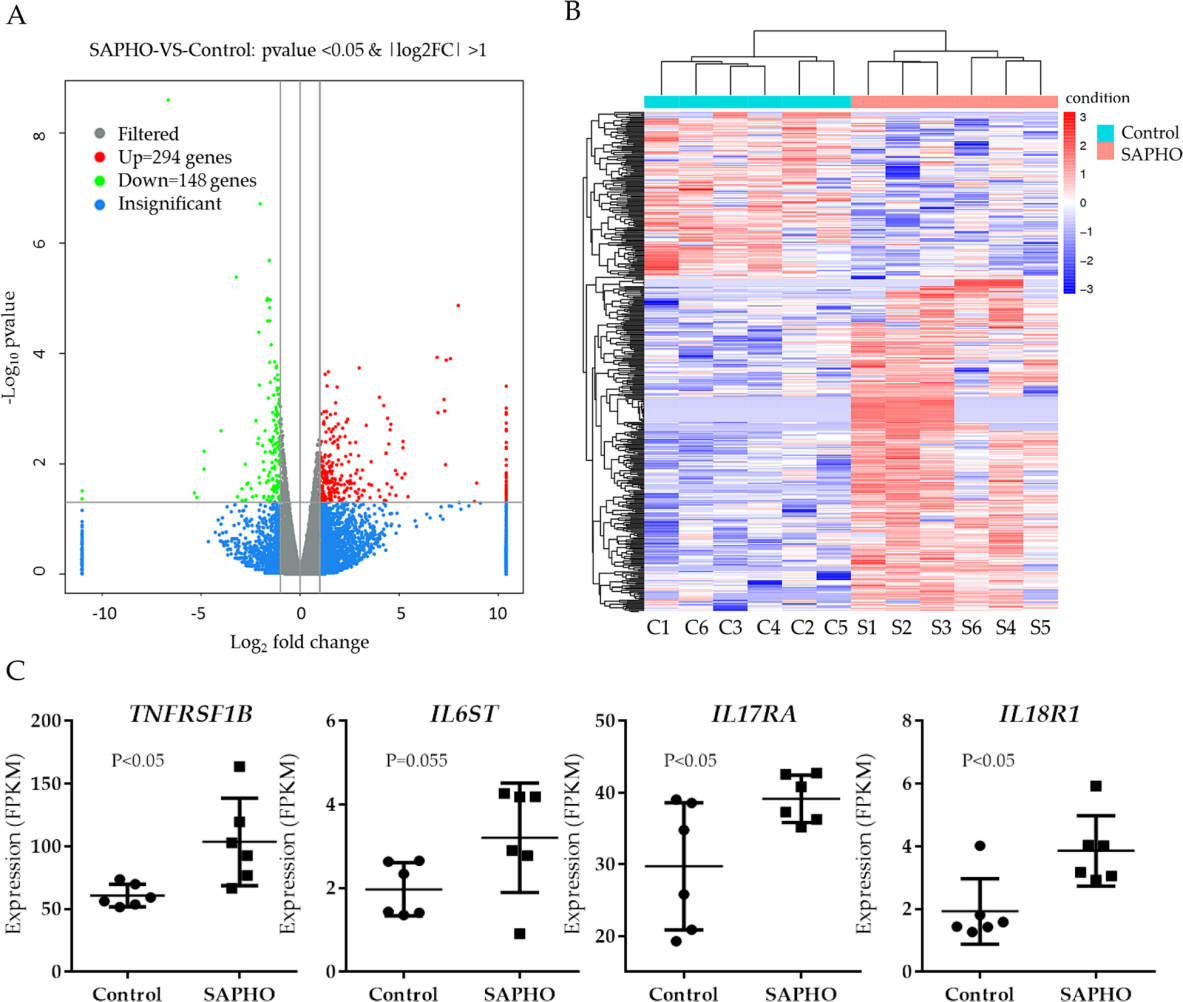
Differentially expressed genes in patients with SAPHO syndrome. **a** Differentially expressed genes were identified using “fold change > 2.00 and *P* value < 0.05” as a cutoff. **b** Hierarchical Clustering analysis of differentially expressed genes in SAPHO patients showed closer relationship within groups. **c** Gene expression (FPKM) of *TNFRSF1B*, *IL6ST*, *IL17RA* and *IL18R1*, *p* value was calculated by Student t-test

The differentially expressed mRNAs were identified between SAPHO group and Control group using “*P* < 0.05, fold change > 2” as a cutoff (Fig. 2a). The Hierarchical Clustering analysis was conducted to illustrate the distinguishable expression pattern between the two groups (Fig. 2b). A total of 442 differentially expressed genes were identified, among which 294 were up-regulated and 148 down-regulated (Additional file 2: Table S2). As reported before, tumor necrosis factor-α (TNF-α) signaling plays important role in the pathogenesis of SAPHO syndrom [18], while IL6, IL17 and IL18 were reported to be elevated in the sera of SAPHO patients [19–21]. We found that TNF-α receptor encoding genes *TNFRSF1B*, IL6 receptor subunit *IL6ST*, IL17 receptor gene *IL17RA* and IL18 receptor genes *IL18R1* were up-regulated in peripheral blood neutrophils of SAPHO patients (Fig. 2c). These results indicated that peripheral neutrophils of SAPHO patients were in a more active status than those of the healthy controls.

### Validation of differentially expressed genes and correlation with disease parameters

**Fig. 3:**
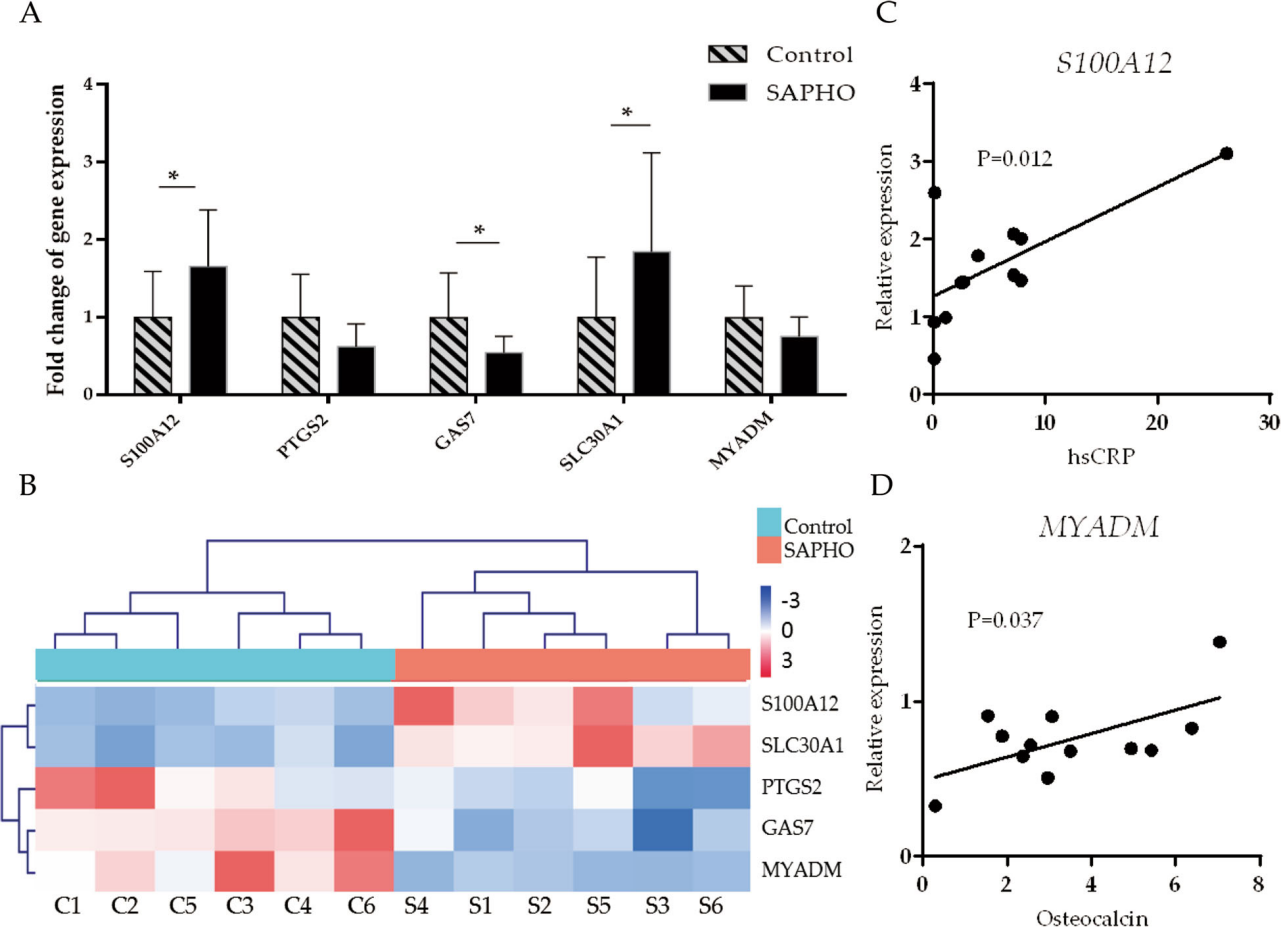
Verification of differentially expressed genes and correlation with disease parameters. **a** relative expression of five genes by qRT-PCR in 12 patients and 12 healthy controls, *: *P* < 0.05 **b** HCL cluster of FKPM of 5 verified genes based on RNA-Seq data **c** correlation analysis between *S100A12* and hsCRP **d** correlation analysis between *MYADM* and osteocalcin

Five differentially expressed genes of interest were selected for the validation by qRT-PCR (Fig. 3a) in a larger population (12 patients and 12 gender-age matched healthy controls). Among them, S100 Calcium Binding Protein A12 (S100A12) has been detected in salivary proteome profile of SAPHO syndrome [22]. Prostaglandin-endoperoxide synthase 2 (*PTGS2* or *COX-2*) was down-regulated in chronic inflammation [23], whose inhibitors are considered as a potential risk factor for bone healing [24]. In the meanwhile, growth-arrest-specific 7 gene (*GAS7*) is required for osteoblast differentiation [25], which may play a role in bone manifestations in SAPHO syndrome. Besides, a feedback loop between inflammation and Zn uptake has been observed in rheumatoid arthritis [26], while Zn transporters, also known as SLC30 families, are related to different skin disorders [27] as well as systemic inflammatory response syndrome [28]. Besides, myeloid-associated differentiation marker (*MYADM)* drew our attention because of its important role in cell spreading and migration [29], which is consistent with our findings in GO term enrichment analysis. The expression levels of *S100A12* and *SLC30A1* were up regulated while those of *PTGS2*, *GAS7* and *MYADM* were down-regulated in SAPHO patients, which was in accordance with the RNA-Seq data (Fig. 3b). However, not all the differences in gene expression were statistically significant, and the verification in a larger population is still necessary. To further investigate the biological significance of differentially expressed genes, their correlation with clinical parameters was analyzed. Interestingly, a positive correlation was observed between *S100A12* and peripheral hsCRP (Fig. 3c), indicating a role in inflammation. Besides, the down-regulated gene *MYADM* was positively correlated to osteocalcin (Fig. 3d), which is essential for bone metabolism.

### KEGG pathway enrichment of differentially expressed genes

**Fig. 4:**
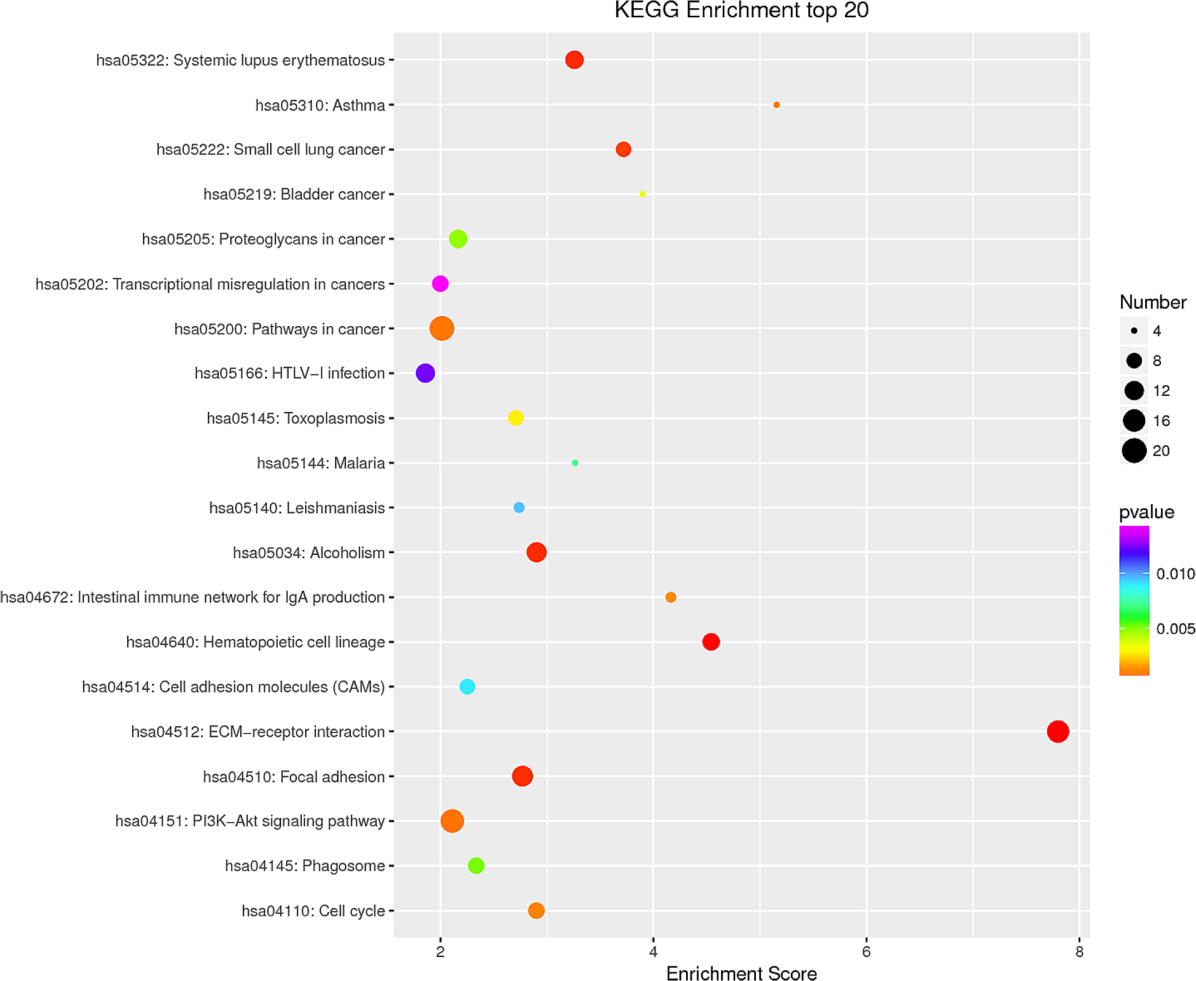
Scatter plot for top 20 pathways in KEGG enrichment of differentially expressed genes. The enrichment score was calculated according to the number of annotated genes and that of all annotated genes in this pathway term, see method in detail. Lower *P*-values indicate higher pathway enrichments

Kyoto Encyclopedia of Genes and Genomes (KEGG) analysis showed that differentially expressed genes were mainly enriched in pathways about autoimmunity, infection, cancer and metabolism (Fig. 4). Among them, the involvement of system lupus erythematosus (SLE) drew our attention. Similar to SAPHO syndrome, SLE is an autoimmune disease with diverse presentations ranging from rash and arthritis to anemia, serositis, nephritis, seizures, etc., while incidence of both diseases is higher in women aged 15–50 [30]. Anti-nucleosome antibodies as well as anti-dsDNA antibodies are present and essential in the diagnosis of patients with SLE [31], while mRNAs encoding histone-derived peptides such as *H2A*, *H2B*, *H3* and *H4* were up-regulated in SAPHO group. The activation of SLE pathway in patients may implicate an immune-mediated etiology in SAPHO syndrome. Besides, extracellular matrix (ECM)-receptor interaction is also among the top 20 significant pathways, which was commonly associated with poor prognosis of cancer [32], and recent study also revealed its association with osteoarthritis [33]. Genes enriched in each pathway are shown in Additional file 3: Table S3.

### Functional prediction of differentially expressed genes in GO database

**Fig. 5:**
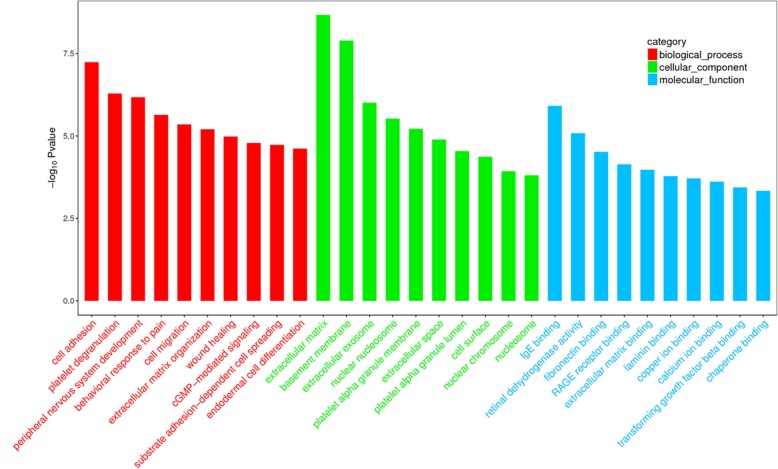
Top 10 GO terms (BP, CC, MF) associated with differentially expressed genes. Top 10 significant GO terms in biological process are shown in red, while those in cellular component in green and molecular function in blue

The top 10 significantly enriched terms of biological process, cellular component and molecular function in Gene Ontology (GO) analysis are shown in Fig. 5. In terms of biological process, differentially expressed genes were predicted to be involved in GO terms such as cell adhesion, platelet degranulation and peripheral nervous system development. The most significantly indicated terms were extracellar matrix in cell component and IgE binding in molecular function. Interestingly, differentially expressed genes were enriched in cell migration (14 genes enriched) and cell adhesion (28 genes enriched), which were both important for neutrophils recruitment in response to inflammation. Genes enriched in each GO term are shown in Additional file 4: Table S4.

### PPI network among genes enriched in cell adhesion and migration

**Fig. 6:**
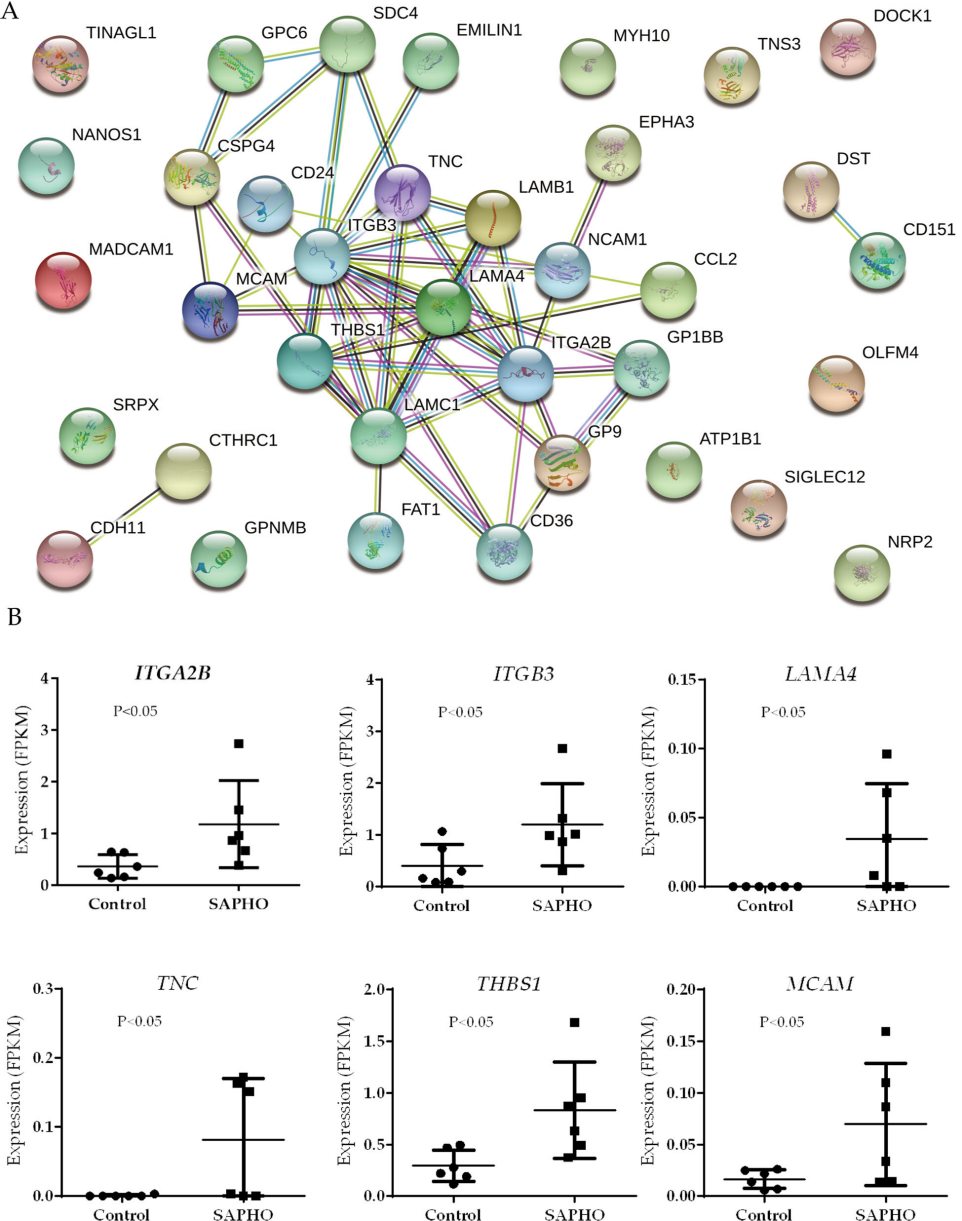
PPI network and expression of hub genes enriched in cell adhesion and migration **a** PPI network of genes enriched in cell adhesion and migration **b** Expression level of hub genes in the PPI network based on RNA-Seq data

To gain insight into how neutrophil adhesion and migration are regulated in patients with SAPHO syndrome, the network of protein-protein interaction (PPI) among genes enriched in cell adhesion and cell migration was constructed based on STRING database (Fig. 6a). The up-regulation of hub genes in the network indicated an over-activated cell adhesion and migration in patients with SAPHO syndrome (Fig. 6b). Among them were components of laminin (*LAMA4*, *LAMB1* and *LAMC1*) and melanoma cell adhesion molecule (*MCAM*), which functions as a receptor for *LAMA4* [34], promoting cell adhesion and slow-rolling of neutrophils [35]. Up-regulation of tenascin-C (*TNC*), an endogenous ECM molecule required in neutrophil recruitment [36], was also observed in SAPHO group.

### Enhanced neutrophil recruitment in SAPHO patients

**Fig. 7:**
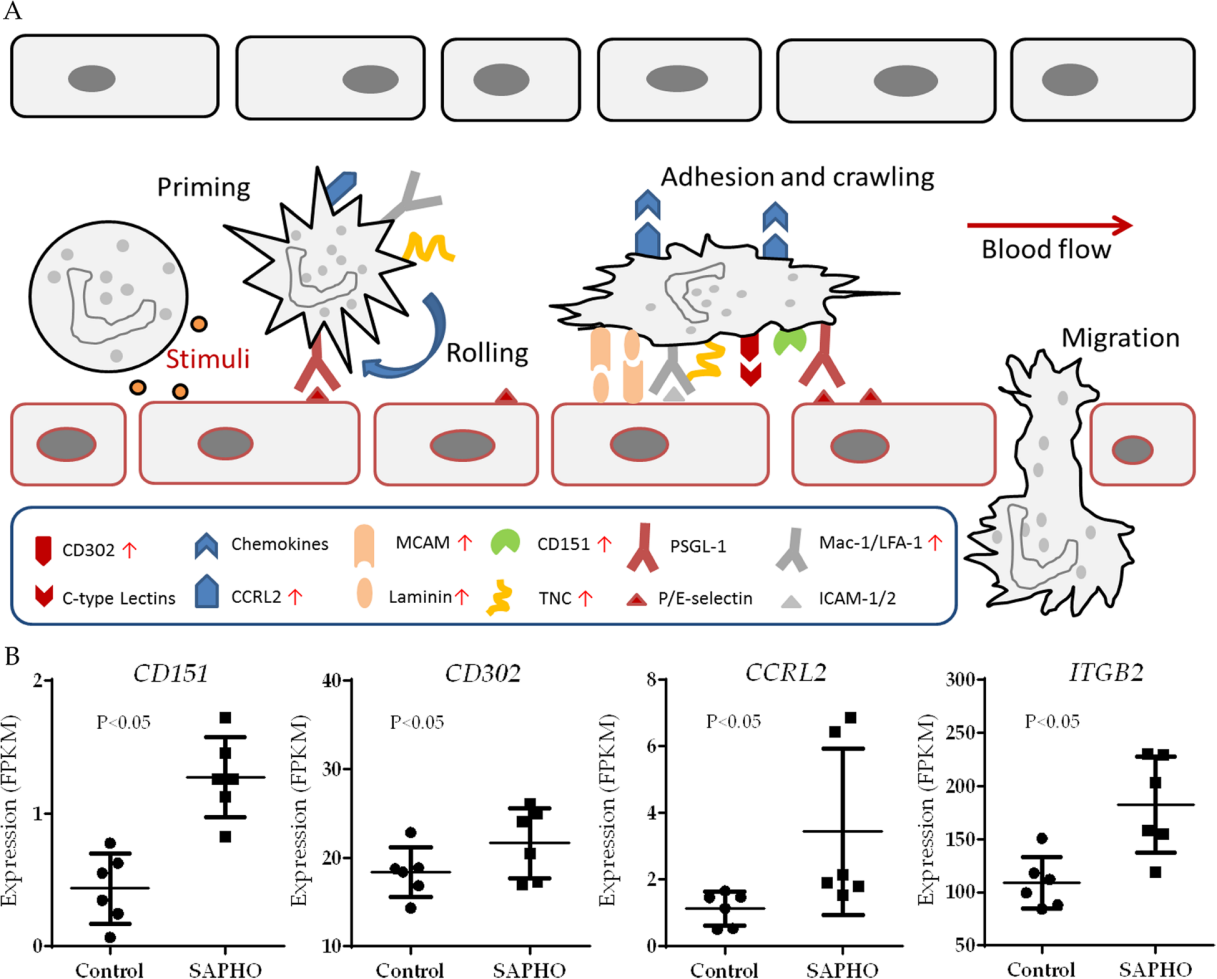
Enhanced neutrophil recruitment in patients with SAPHO syndrome **a** Neutrophils in peripheral blood are primed by stimuli. Active transcription and expression of receptors or other molecules are then initiated, while the endothelial cells present chemokines which mediate neutrophil arrest, rolling, adhesion, crawling and migration. Differentially expressed genes in SAPHO group are marked with a red arrow. **b** Expression levels of up-regulated genes participating in the process of neutrophil recruitment based on RNA-Seq data, *p* value was calculated by Student t-test

As previously described, the recruitment of neutrophils consists of priming, rolling, arrest, adhesion, crawling, and migration [37]. Specifically, resting neutrophils in peripheral blood are primed by stimuli such as cytokines and interaction with activated endothelial cells, which leads to active transcription and expression of receptors or other molecules [38], among which the P-selectin glycoprotein ligand 1 (*PSGL-1*) expressed by neutrophils binds in high on-and-off-rate to P-selectins on the endothelial cells and mediates the rolling step [39]. Afterwards, the endothelial cells present chemokines which activate lymphocyte function-associated antigen-1 (LFA-1) and macrophage-1 antigen (Mac-1) on neutrophils, promoting neutrophil arrest [40]. Neutrophils then crawl along the surface of the endothelium to a suitable extravasation site (Fig. 7a). Besides, as we further scanned the differentially expressed genes, several additional genes were found to play important roles in neutrophil recruitment, including the up-regulated gene *ITGB2,* which is component of LFA-1 and Mac-1. Another increased gene, C-C chemokine receptor-like 2 (*CCRL2*), has also been proved to be crucial in neutrophil recruitment at sites of inflammation [41]. Other genes such as *CD302*, a C-type lectin receptor involved in cell adhesion and migration [42] and *CD151*, known to complex with integrins and other transmembrane 4 superfamily proteins [43], were also up-regulated in SAPHO syndrome (Fig. 7b).

## Discussion

SAPHO syndrome is a chronic and recurrently disease characterized by cutaneous and osteoarticular inflammation. Both diagnosis and treatment for this rare disease have been limited by its heterogeneous manifestation and elusive etiology. Circulating neutrophils are very sensitive to inflammatory signals and are the first pioneers recruited to the inflammatory site [13]. Besides, enhanced neutrophil infiltration was observed in mice with a SAPHO syndrome-like phenotype [10]. In this study, RNA-Seq was performed to profile the mRNA expression level of neutrophils purified from peripheral blood of SAPHO patients. A total of 442 differentially expressed genes were identified (294 upregulated and 148 downregulated). It has been reported that TNF-α signaling pathway plays an important role in the pathogenesis of SAPHO [18], while IL6, IL17 and IL18 were elevated in the serum of SAPHO patients [19–21]. Genes encoding the receptors for TNFα, IL6, IL17 and IL18 were up-regulated in peripheral blood neutrophils of SAPHO patients. KEGG analysis showed that differentially expressed genes were enriched in “systemic lupus erythematosus” and “ECM-receptor interaction”. GO enrichment suggested that differentially expressed genes may function by participating in biological processes such as “cell migration” and “cell adhesion”. These results indicated that the peripheral blood neutrophils in SAPHO patients might have enhanced activation and migration potential.

Five differentially expressed genes of interest were validated by qRT-PCR, among which *S100A12* was observed to be elevated in the saliva of SAPHO patients [22] and serum *S100A12* is related to juvenile idiopathic arthritis-associated uveitis [44]. Besides, *S100A12* secreted by neutrophils is a regulator of reactive oxygen species and potassium efflux [45]. *PTGS2* was down-regulated in SAPHO patients, which was also previously reported in chronic inflammation [23]. As *PTGS2* inhibitors are considered to be a potential risk factor for bone healing [24], we suspect that the down-regulation of *PTGS2* in SAPHO syndrome may contribute to the osteoarticular lesions in patients. The results of qRT-PCR were approximately in consistence with RNA-Seq data. *S100A12* was elevated in neutrophils from patients with SAPHO syndrome in our study, and the expression level is positively related to serum hsCRP. Meanwhile, the down-regulated gene *MYADM* was positively related to serum osteocalcin in SAPHO patients. Osteocalcin is mainly expressed by osteoclast and plays a key role in bone endocrine functions [46]. The down-regulation of *MYADM* may contribute to bone metabolism and delayed bone recovery in SAPHO syndrome. Thus, these genes may serve as potential markers of disease activity in clinical treatment instruction. However, the mechanism how these genes participated in the etiology and development of SAPHO syndrome requires further investigation.

To gain insight into the characterization of biological functions of differentially expressed genes between SAPHO group and the control group, GO term enrichment and KEGG pathway analysis were also performed. Eleven of the differentially expressed genes were enriched in SLE pathway, indicating that an autoimmune dysfunction may be involved in SAPHO syndrome. Pathologically, the histone-derived peptides H2B_10–33_, H4_16–39_, H4_71–94_, H3_91–105_, H2A_34–48_, and H4_49–63_ stimulated T cells from patients with lupus to produce cytokines, which can help B cells respond to antigenic epitopes of nucleosomes. Meanwhile, the role of neutrophils in SLE has been indicated as the proteins derived from neutrophil extracellular traps (NETs) may serve as autoantigens [47] and that infiltrating neutrophils in skin and kidney contribute to the pathology of SLE by undergoing NETosis and releasing nucleic acids and proteins [48]. We found that mRNAs of *H2A*, *H2B*, *H3* and *H4* are also up-regulated in neutrophils of SAPHO patients, which indicated that neutrophils of SAPHO patients may be prone to NETosis.

The role of neutrophil migration in inflammation has drawn increased interest in the past decade and recent evidences suggested that complex neutrophil behavior of forward and reverse migration contribute to resolution of inflammation [49]. In this study, 28 of the differentially expressed genes were enriched in the GO term of cell adhesion and 14 in cell migration. Based on the STRING database, we identified the PPI network of these genes and several hub regulators in the biological process. The up-regulation of hub genes in the network indicated an over-activation of cell adhesion and cell migration in SAPHO syndrome. Specifically, resting neutrophils are primed by inflammation and active transcription of genes such as *TNC*, *CCRL2* and *ITGB2* (CD18) are initiated. The up-regulated *ITGB2* is component of LFA-1 and Mac-1, which interact with ICAM on endothelial cells, promoting neutrophil arrest [40]. Components of laminin (*LAMA4*, *LAMB1* and *LAMC1*) and *MCAM* also help cell adhesion and slow-rolling of neutrophils [35], the up-regulation of whom indicated excessive neutrophil activation and migration in SAPHO syndrome. Other up-regulated genes such as *CD302* and *CD151* were also reported to play important roles in cell adhesion and migration [42, 43]. Thus, we speculated that over-active neutrophil adhesion and migration may contribute to the pathology of SAPHO syndrome, which is in consistence with the excessive neutrophil infiltration on multiple organs in SAPHO patients.

## Conclusion

In summary, our research provided the first insight into peripheral blood neutrophil mRNA profile for SAPHO patients, offering potential biomarkers for disease diagnosis and prognosis. We found that enhanced migration and adhesion of peripheral blood neutrophils may play important roles in the pathogenesis of SAPHO syndrome, which indicated that inhibition of neutrophil migration and adhesion might be a candidate therapeutic strategy for SAPHO syndrome.

## Materials and methods

### Subjects and samples

This study was approved by the Institutional Review Board of Peking Union Medical College Hospital (protocol number: ZS-994). We recruited 12 patients with SAPHO syndrome (informing consent obtained) between March 2017 and June 2018. As controls, we included 12 healthy volunteers in the same gender and age range. 20 ml peripheral blood sample was collected from each participant and neutrophils were purified with Polymorphprep™ by gradient centrifugation (neutrophil purities > 90%).

### Sequencing and analysis of RNA expression

The total RNA was isolated from neutrophils using Trizol reagent (Invitrogen Life Technologies, Carlsbad, CA, USA). Qualified RNA (rRNA removed) was reverse transcribed into cDNA after fragmentation. RNA sequencing was constructed on Illumina HiSeq 2000 Platform. Clean data was mapped to the reference database GRCh38 with HISAT2 [17], while Bowtie2 [50] and eXpress [51] were used to assemble the transcripts. The expression level of RNA was calculated by fragments per kilobase of exon per million fragments mapped (FPKM). Differentially expressed mRNAs between Control group and SAPHO group were determined by DEseq [52] with “*p* < 0.05, fold change > 2” as the threshold for significance.

### Quantitative real-time PCR

Relative expression levels of selected genes were determined by quantitative real-time PCR (qRT-PCR). 200–500 ng of total RNA was reverse transcribed to cDNA using a qPCR RT Master Mix with gDNA Remover Kit (TOYOBO). QRT-PCR was performed on Biorad iQ5 machine using a SYBR Green Realtime PCR Master MixPremix (TOYOBO) according to the manufacturer’s instruction. All experiments were conducted in three technical replicates. The relative expression level of each target gene was normalized to the expression of β-actin with the 2^−ΔΔCt^ method. Primers are shown in Additional file 5: Table S5.

### GO term enrichment and KEGG pathway analysis

Differentially expressed genes were selected for functional annotation. Terms of biological process, cellular component and molecular function were analyzed according to Gene Ontology (GO) database (http://amigo.geneontology.org/amigo/landing) and functional pathway analysis were based on Kyoto Encyclopedia of Genes and Genomes (KEGG) database (http://www.kegg.jp/). Hypergeometric distribution test was used to determine the significance of differentially expressed mRNA enrichment in each GO term or KEGG pathway. The enrichment score was evaluated as follows:

$$ Enrichment\ score=\raisebox{1ex}{$\frac{m\ }{n\ }$}\!\left/ \!\raisebox{-1ex}{$\frac{M}{N}$}\right. $$

Here, N is the number of GO-annotated or KEGG-annotated mRNAs in all genes, while n is the number of GO-annotated or KEGG-annotated mRNAs in differentially expressed genes in N. M is the number of mRNAs annotated to a particular GO term or KEGG pathway in all genes while m is the number of genes annotated to a particular GO term or KEGG pathway in differently expressed genes.

### Statistical analysis

Continuous variables were presented as means ± standard deviation. Student’s t-test was performed using SPSS version 20.0 software for comparisons between groups. The correlation of gene expression and clinical parameters was performed using Pearson’s correlation coefficient. *P* value < 0.05 was considered statistically significant.

## Additional files

Additional file 1: **Table S1.** RNA-Seq data summary. Amount of reads aquired by RNA-Seq and quality control data summary of each sample. (XLSX 10 kb)

Additional file 2: **Table S2.** Differentially expressed genes. List of differentially expressed genes in SAPHO syndrome, related *P* value, fold change, and up/down regulation of genes were shown on Additional file 1: Table S1. (XLSX 37 kb)

Additional file 3: **Table 3.** Genes enriched in top 20 KEGG pathways. *P* value, enrichment score, and list of genes enriched in each particular pathway of the top 20 KEGG pathways involved in SAPHO syndrome. (XLSX 11 kb)

Additional file 4: **Table S4.** Genes enriched in top 10 GO terms of BP,CC,MF. *P* value, enrichment score, and list of genes enriched in each GO term of the top 10 GO terms of BP,CC,MF involved in SAPHO syndrome. (XLSX 14 kb)

Additional file 5: **Table 5.** Primers of differentially expressed genes. Sequence of foward and reverse primers used in the qRT-PCR verification of differentially expressed genes. (XLSX 9 kb)

## Data Availability

Original data of RNA-Seq has been submitted to www.ncbi.nlm.nih.gov/sra/. Data and materials are available from corresponding authors on reasonable request.

## Abbreviations

CCRL2: C-C chemokine receptor-like 2
ECM: Extracellular metrix
ESR: Erythrocyte sedimentation rate
FPKM: Fragments per kilobase of exon per million fragments mapped
GAS7: Growth-arrest-specific 7
GO: Gene Ontology
hsCRP: High sensitivity C-Reactive protein
IBD: Inflammatory bowel disease
ITGB2: Integrin beta 2
KEGG: Kyoto Encyclopaedia of Genes & Genomes
LFA-1: Lymphocyte function-associated antigen-1
Mac-1: Macrophage-1 antigen
MCAM: Melanoma cell adhesion molecule
MYADM: Myeloid-associated differentiation marker
NETs: Neutrophil extracellular traps
PCA: Principal component analysis
PMA: Phorbol myristate acetate
PPI: Protein-protein interaction
PSGL-1: P-selectin glycoprotein ligand 1
PTGS2: Prostaglandin-endoperoxide synthase 2
RNA-Seq: RNA sequencing
S100A12: S100 Calcium Binding Protein A12
SAPHO: Synovitis, Acne, Pustulosis, Hyperostosis and Osteitis
SLE: System lupus erythematosus
TNC: Tenascin-C
TNF-α: Tumor necrosis factor-α
VAS: Visual analogue scale
